# Post-adenoviral parasympathetic dysautonomia in a child: a case report

**DOI:** 10.1186/1752-1947-4-182

**Published:** 2010-06-18

**Authors:** Terence F McLoughlin, Adalaida Martinez

**Affiliations:** 1 Newham University Hospital NHS Trust, Glen Road, Plaistow, London, E13 8SL, UK; 2 Barts and the Royal London NHS Trust, Whitechapel Road, Whitechapel, London, E1 1BB, UK

## Abstract

**Introduction:**

This is the first reported case of adenoviral meningoencephalitis that was complicated by persistent parasympathetic dysautonomia, which clinically either stimulated or inhibited its activity.

**Case presentation:**

A 7-year-old Caucasian girl presented to our hospital in March 2008 with a three day history of upper respiratory infection. Her condition worsened and she was placed on ventilator support for two weeks. Her recovery was complicated by a persistent selective parasympathetic dysautonomia. Her past medical history was unremarkable.

**Conclusions:**

To the best of our knowledge this is the first case of an adenoviral infection in a child which was complicated after recovery from an acute meningoencephalitis and peripheral neuropathy.

## Introduction

Neurological complications are uncommon sequelae of adenovirus infection. To the best of our knowledge this is the first reported case of a child whose recovery from adenoviral meningoencephalitis was complicated by persistent dysautonomia confined to the parasympathetic nervous system and which appeared clinically to either stimulate or inhibit its activity.

## Case presentation

Our patient, a 7-year-old Caucasian girl, was admitted to the children's hospital with a three day history of upper respiratory tract infection. Her breathing had become labored and, when seen in the Acute Admission Unit, her pO_2 _saturation fluctuated between 90 and 92% which was refractory to supplementary oxygen. She was placed on ventilator support. An attempt was made to "wean" her off the ventilator after 48 hours. She then exhibited clinical features of cerebral irritation including photophobia and a positive Kernig's sign. Gadolinium-enhanced magnetic resonance imaging (MRI) of her brain confirmed the diagnosis of meningoencephalitis (post-contrast *T*_1_-weighted image indicated an abnormal meningeal enhancement in both hemispheres). Adenovirus serotype 26 was isolated from bronchial aspirate and cerebrospinal fluid (CSF; CSF results: appearance, clear, protein, 0.65 g/L; glucose, 70% of plasma glucose; cell count, lymphocytic. Additional CSF virology, adenovirus serotype 26).

After two weeks it was possible to withdraw ventilator support. She was found to have a fixed dilated left pupil and grade 2/5 weakness of upper and lower limb flexor and extensor muscle groups. Nerve conduction studies confirmed isolated F-wave absence with prolonged distal latencies. A Landry-Guillain-Barre variant was considered to be part of the differential diagnosis. Anti-ganglioside antibodies were negative. A month later muscle weakness was grade 4/5 and nerve conduction studies had reverted to normal. She continued to exhibit signs of a selective parasympathetic dysautonomia which included: a fixed dilated left pupil; profound bradycardia; hyper-salivation; fore- and hindgut dysmotility with severe dysphagia and reflux together with anorectal and urinary overflow incontinence.

Her past medical history, neonatal developmental milestones, family, drug and social histories were unremarkable. All courses of recommended immunization had been completed.

Fifteen months after the acute myeloencephalitic stage of the illness she had made a complete neurological recovery. Our patient was lost to follow-up.

## Discussion

The patient presented with a viral upper respiratory tract infection caused by adenovirus serotype 26. She went on to develop meningoencephalitis. The same virus was isolated from the CSF. Adenoviruses are a common cause of childhood infection [1] with protean presentation. The portals of entry are the respiratory and feco-oral routes. The peak incidence in the UK of adenovirus infection is in mid-winter. Our patient was admitted in January. Polyradiculopathy is the established cause of the Landry-Guillain-Barre syndrome (LGBS) [2]. LGBS was first reported in association with confirmed adenoviral infection by Wells [3]. Ileocaecal intussusception in childhood is a described complication of a post-adenoviral gastrointestinal infection. It is speculated that the pathogenesis is a mesenteric parasympathetic inflammatory neuropathy at the junction of the vagal and sacral parasympathetic innervation of the gut [4]. The clinical presentation of a post-viral neuropathy will depend upon which nerve fibers are affected by the viral demyelinating pathology. In our case following recovery of the adenoviral meningoencephalitis and peripheral neuropathy the adenoviral neuropathic effect persisted but was confined to the parasympathetic fibers of the cranial and peripheral nerves.

Improvement in the meningoencephalitic phase of the illness left her with a peripheral neuropathy which finally resolved within a month. During and after this time she exhibited symptoms and signs of parasympathetic neuronal involvement. Table 1 lists how parasympathetic involvement depending upon which efferent fibers that had been attacked by the virus either stimulated [CN VII and IX] or inhibited [CN III and X and the nervi errigentes S2, 3 and 4] parasympathetic activity and more likely than not accounted for the corresponding symptoms and physical signs.

**Table 1:** Adenoviral cranial and peripheral nerve involvement causing parasympathetic dysautonomia.

Symptoms and/or signs	PS involvement	Nerve(s) affected
Fixed dilated left pupil	PS motor paralysis	Left CN III
Hypersalivation	PS secretomotor	CN VII/IX
Foregut dysmotility with:	PS efferent	CN X
a) Dysphagia		
b) Gastrostasis		
c) Duodenal ileus		
Bradycardia	PS efferent	CN X
Hindgut dysmotility:	PS efferent	S 2, 3, 4

The fixed dilated left pupil was the last physical sign to resolve. It was not possible to establish whether this represented continuing post-meningoencephalitic damage to the Edinger-Westphal nucleus [5] or damage to the parasympathetic outflow in CN III.

She hyper-salivated. The likely explanation for this was viral stimulation of the parasympathetic secretomotor fibers in CN VII (submandibular and sublingual glands) and CN IX (the parotids).

The patient had a profound sinus bradycardia. Viral damage to the vagus more likely than not stimulated CN X fibers thus increasing vagal "tone".

Vagal damage would also explain the symptoms of foregut dysmotility causing dysphagia, delayed gastric emptying and reflux.

The patient was doubly incontinent and had an absent anal reflex. The S2, 3, and 4 roots contain the nervi errigentes parasympathetic motor supply to the pelvic viscera. Viral damage to the nervi errigentes would cause fecal and urinary overflow incontinence.

## Conclusions

Autonomic dysfunction is well known to be associated with polyneuropathies [6]. To the best of our knowledge this is the first reported case of an adenoviral infection in a child which was complicated after recovery from acute meningoencephalitis and peripheral neuropathy by a continuing selective parasympathetic dysautonomia which either caused selective stimulation or inhibition.

## Abbreviations

CN: cranial nerve; S: sacral nerve; PS: parasympathetic; Please note that cranial nerves are traditionally described using Roman numerals and sacral nerves are described using numbers.

## Competing interests

The authors declare that they have no competing interests.

## Authors' contributions

TM was responsible for patient care, case analysis, drafting and revising the manuscript. AM carried out the literature review, drafting and revising the manuscript. All authors read and approved the final manuscript and participated in this case study.

## Consent

Written informed consent was obtained from the patient's parents for publication of this case report and accompanying table. A copy of the written consent is available for review by the Editor-in-Chief of this journal.
